# The cAMP signaling pathway mediates photoperiod-induced follicle development in striped hamsters (*Cricetulus barabensis*) supported by association analyses

**DOI:** 10.3389/fendo.2026.1794734

**Published:** 2026-04-16

**Authors:** Huiliang Xue, Xueting Zhang, Wen Qi, Chao Fan, Jinhui Xu, Lei Chen, Ming Wu, Laixiang Xu

**Affiliations:** 1 College of Life Sciences, Qufu Normal University, Qufu, Shandong, China; 2 Shandong Key Laboratory of Wetland Ecology and Biodiversity Conservation in the Lower Yellow River, Qufu, Shandong, China

**Keywords:** cAMP signaling pathway, follicle development, photoperiod, seasonal reproduction, striped hamsters

## Abstract

**Background:**

Rodents represent one of the key functional groups in ecosystems, and their population outbreaks can disrupt ecological equilibrium and cause substantial economic losses in agricultural production. Therefore, rational control of rodent populations is essential for maintaining ecosystem stability and minimizing economic damage. The striped hamster displays marked seasonal reproductive patterns, leading to significant fluctuations in population size across seasons. Investigating how female striped hamsters regulate follicle development in response to photoperiodic cues offers a promising target for the strategic management of pest populations.

**Methods:**

Adult female striped hamsters were exposed to long (LP), medium (MP), and short (SP) photoperiods. Ovarian follicle development was assessed through hematoxylin and eosin (HE) staining of ovaries. Transcriptome sequencing was conducted to identify signaling pathways associated with photoperiodic regulation of follicle development. Serum levels of FSH, LH, and cAMP, as well as ovarian cAMP concentrations, were measured using enzyme-linked immunosorbent assays (ELISA). Real-time quantitative PCR (qPCR) was further employed to quantify the expression of key genes involved in follicle development. Correlations between cAMP levels and hormonal or gene expression data were analyzed to elucidate the main factors mediating photoperiod-regulated follicle development.

**Results:**

Long photoperiod (LP) promotes follicle development, whereas short photoperiod (SP) suppresses it. The cAMP signaling pathway serves as a key mechanism mediating photoperiod to regulate follicle development. Photoperiod modulates the synthesis of FSH, LH, and cAMP, thereby coordinately influencing downstream reproductive physiology.

**Conclusions:**

GPR3, ADCY5, PDE1A, and PDE3A are identified as core factors in the cAMP signaling pathway and are proposed as potential molecular targets for the rational control of farmland rodent populations.

## 1 Introduction

Reasonable control of animal population abundance is of great significance for maintaining ecosystem balance and minimizing economic losses. Some animal species restrict their reproductive activities to specific seasons (1), thereby ensuring that offspring are born during the most favorable periods, which enhances the survival rate of the next generation and supports the long-term stability of the population. Photoperiod serves as a critical environmental cue that regulates the timing of seasonal reproductive activities in animals (2). This regulatory effect is mediated through the hypothalamic-pituitary-gonadal axis (HPGA) (3). Light signals, upon reception by the animal retina, are transformed into neural impulses and subsequently transmitted to the suprachiasmatic nucleus of the hypothalamus and the pineal gland. The pineal gland then converts these neural signals into endocrine signals through the synthesis and secretion of melatonin (MT). Melatonin modulates the secretion of gonadotropin-releasing hormone (GnRH), thereby regulating the synthesis and release of gonadotropins from the pituitary gland. These hormones are then transported via the bloodstream to the gonads, where they exert their effects and participate in the regulation of reproductive functions (4, 5). Research indicates that exposure to short photoperiods induces gonadal atrophy in rodents (6), disrupts follicle development, and markedly decreases the number of antral follicles and corpora lutea. Notably, when Siberian hamsters are transitioned from a short to a long photoperiod, follicle development is re-established and ovarian function recovers (7, 8). These findings provide further evidence that photoperiod serves as a primary environmental cue governing seasonal reproduction in animals.

The ovary serves as a critical reproductive organ in female mammals (9). Follicles are classified into distinct developmental stages—primordial follicles, primary follicles, secondary follicles, antral follicles, and mature follicles (10). The specific stages of follicle development are detailed in Xue et al. (11). The developmental status of ovarian follicles is a critical determinant of reproductive activity intensity. For example, in female animals with polycystic ovary syndrome (PCOS), disruptions in early follicle development result in anovulatory infertility (12). Therefore, investigating the mechanisms through which photoperiod regulates follicle development may provide valuable insights for managing animal population dynamics.

Photoperiod-regulated endocrine signals and follicle microenvironmental cues coordinately govern follicle development (13, 14). Cyclic adenosine monophosphate (cAMP) functions as a central intracellular second messenger that transduces extracellular signals into specific cellular responses (15) and engages multiple effector proteins to orchestrate distinct signaling pathways governing key biological processes, including metabolism, gene expression, and neurotransmitter synthesis (16). During female reproduction, luteinizing hormone (LH) binds to its G protein-coupled receptor on ovarian cells to drive cAMP synthesis, a key second messenger pathway essential for coordinating follicle maturation and the resumption of oocyte meiosis (17). Supplementing the culture medium of rat ovaries explants with FSH enhances phosphodiesterase (PDE) activity, leading to accelerated cAMP degradation and a subsequent decrease in intracellular cAMP levels (18). Elevated levels of cAMP in the fetal ovary inhibit primordial follicle formation (19), whereas increased cAMP levels in the ovaries of neonatal mice promote the activation of these follicles (20). Therefore, it is hypothesized that LH and FSH may regulate cAMP levels through modulation of upstream regulatory factors, inactivation of adenylyl cyclase, or activation of cAMP-degrading enzyme activity, thereby influencing downstream signaling pathways and ultimately affecting follicle development.

Studying the mechanism by which cAMP regulates follicle development is conducive to the rational control of follicle development (21), and thereby influencing the population size of animals. G protein-coupled receptors (GPCRs) are integral membrane proteins localized on the cell surface (22). GPCRs transduce extracellular signals into intracellular responses and modulate the intracellular concentration of cAMP. A variety of G proteins regulate cAMP signaling *in vivo*, including stimulatory G proteins (Gs), which activate adenylyl cyclase (ADCY), and inhibitory G proteins (Gi), which suppress ADCY activity (23–25). G protein-coupled receptor 3 (GPR3) and G protein-coupled receptor 12 (GPR12) are members of the orphan G protein-coupled receptor superfamily and exhibit a high degree of sequence homology (26, 27). The cAMP level in mouse and rat oocytes is determined by the expression levels of GPR3 and GPR12 (28), and female mice lacking GPR3 exhibit infertility (29). The concentration of cAMP in ovarian tissues is also determined by the balance between its synthesis and degradation rates. Adenylyl Cyclase (ADCY) is the key enzyme responsible for cAMP synthesis, catalyzing the conversion of adenosine triphosphate (ATP) into cAMP by removing one pyrophosphate. Adenylyl Cyclase (ADCY) can be categorized into six distinct classes (types I–VI) based on structural characteristics and localization (30). Among these, ADCY3 exhibits the highest expression level in mouse oocytes (31), and plays a regulatory role in cAMP synthesis within the ovaries of neonatal mice (20). ADCY3, ADCY5, and ADCY9 exhibit high expression levels in human primordial and primary follicles. Phosphodiesterases (PDEs) are capable of hydrolyzing cAMP into 5′-adenosine monophosphate (5′ -AMP). In mammals, the PDE family is classified into 11 types based on substrate specificity, structural characteristics, and enzymatic kinetic properties (32). Among the PDE family members, PDE3A is predominantly expressed in oocytes, where it hydrolyzes cAMP to regulate its intracellular concentration (33). Disruption of PDE3A in female mice results in cell cycle arrest of ovarian oocytes, ultimately leading to infertility (34). PDE3A-deficient mice exhibit impaired cAMP degradation, leading to elevated intracellular cAMP levels and subsequent disruption of meiotic progression and oocyte maturation (29, 35). Downregulation of GPR3 or ADCY3 expression in the ovaries of female mice results in abnormally reduced intracellular cAMP levels in oocytes, accelerated meiotic progression, and ultimately leads to premature ovaries aging (36, 37). In summary, the cAMP signaling pathway regulates follicle development in mammals. It is initiated by extracellular ligands binding to G protein–coupled receptors (GPCRs), leading to activation of adenylyl cyclase (ADCY) to synthesis cAMP, while phosphodiesterases (PDEs) dynamically degrade cAMP to fine-tune intracellular cAMP concentrations, further govern follicle development. Despite its well-established role, the precise molecular mediators, and specific functional outcomes remain incompletely characterized.

The striped hamster (*Cricetulus barabensis*) is a primary agricultural pest rodent distributed in North China. Characterized by its small body size and high reproductive capacity, this species exhibits distinct seasonal breeding patterns, with reproductive activity peaking in spring and autumn and reaching its lowest level during winter. Elucidating the molecular mechanisms by which the cAMP signaling pathway mediates photoperiods to regulate follicle development in striped hamsters facilitates the rational regulation of reproductive activity intensity in animals, thereby enabling effective population management and offering theoretical foundations for maintaining ecological balance and mitigating crop-related economic losses.

## 2 Materials and methods

### 2.1 Sample collection and photoperiod treatments

In this study, adult female striped hamsters were captured from farmland in Wucun Town, Qufu City, Shandong Province, using live-capture cage traps. Following capture, the hamsters underwent surface disinfection and were individually numbered before being housed in animal breeding boxes. The breeding boxes were maintained under natural light conditions, and the striped hamsters had ad libitum access to food and water. The animals were housed in the animal breeding facility at the School of Life Sciences, Qufu Normal University. Prior to the formal photoperiod treatment, adult female striped hamsters approximately 5 months of age, and exhibiting molar wear consistent with age, were selected for the study. The animals were acclimated for two weeks in an animal incubator maintained under a medium photoperiod (MP, 12Light:12Dark). The animal incubator was maintained at a temperature of 22 ± 2°C, relative humidity of 55 ± 5%, and light intensity of 150 ± 10 lx. Following the adaptation period, all animals were reweighed. Thirty-two adult female striped hamsters were randomly assigned to three experimental groups: the long photoperiod group (LP, 16 L:8D, n=13), the short photoperiod group (SP, 8L:16D, n= 14), and the medium photoperiod group (MP, 12L:12D, n=5). Following grouping, no significant differences in body weight were observed among the animals across the groups. The animals were randomly assigned to environmental chambers and maintained under long photoperiod (LP, 16L:8D), medium photoperiod (MP, 12L:12D), and short photoperiod (SP, 8L:16D) conditions for 8 weeks, with light offset synchronized at 18:00 across all three groups. The striped hamsters had ad libitum access to water and were provided with 2–3 g of commercially composite rat food pellets daily (Jinan Peng Yue Experimental Animal Breeding Co., Ltd.).

During the photoperiod treatment, the body weight of the striped hamsters was monitored weekly. After 8 weeks, the estrus status of the striped hamsters was assessed. The striped hamsters determined to be in estrus were humanely euthanized by gradual CO_2_ inhalation. Euthanasia was performed in a dedicated, sound-attenuated chamber (30 cm × 20 cm × 15 cm) initially filled with room air. Medical-grade CO_2_ (density ≈ 1.98 kg/m³) was introduced at a flow rate calibrated to achieve 50-70% chamber volume replacement per minute, ensuring rapid unconsciousness while minimizing aversive stimulation. Loss of consciousness, confirmed by cessation of respiratory effort and fixed, dilated pupils, typically occurred within 90–180 s. If apnea, mydriasis, and absence of heartbeat were not concurrently observed within this timeframe, CO_2_ administration was continued until all three criteria were met, thereby confirming irreversible cessation of vital functions. All procedures were conducted during the animals’ active phase, in a quiet, familiar housing room, to reduce procedural stress and maintain physiological baseline integrity. Fresh blood was collected, allowed to stand at 4°C, and then centrifuged using a high-speed refrigerated centrifuge. The supernatant serum was carefully collected for subsequent hormone level analysis. Three ovaries from each group of the striped hamsters were randomly selected and fixed for paraffin embedding and hematoxylin-eosin (HE) staining. The remaining ovaries and other tissues were immediately frozen in liquid nitrogen and stored at -80°C for subsequent experiments.

Among them, three ovaries from LP and SP group respectively were used for transcriptome sequencing, while the ovaries of the remaining animals in each group were utilized for the measurement of cAMP levels and the expression levels of cAMP pathway-related factors at mRNA level. All experimental procedures were reviewed and approved by the Biomedical Ethics Committee of Qufu Normal University and carried out in strict accordance with the guidelines issued by the Chinese Experimental Animal Ethics Committee.

### 2.2 Paraffin embedding and hematoxylin-eosin staining

The left ovaries of three striped hamsters were selected from each group, and their ovaries were weighed and immediately fixed in 4% paraformaldehyde solution for 24 hours. After thorough fixation, the ovaries were processed through dehydration, clearing, wax immersion, and embedding to obtain paraffin blocks. The paraffin blocks were sectioned continuously using a microtome. Continuous sections approximately 5 µm thick were prepared. One section was selected from the middle region of the ovary, and one additional section each from the upstream and downstream directions, spaced at intervals of five consecutive sections. The sections were dewaxed, rehydrated, stained with hematoxylin and eosin, dehydrated, and mounted with neutral resin to prepare HE-stained sections. A total of three representative sections per ovary were examined under a microscope and photographed to quantify the number of follicles at various developmental stages, measure oocyte diameters, and assess the thickness of granulosa cell layers in the ovaries of striped hamsters under different photoperiods. Oocyte diameter was determined as the mean of two perpendicular measurements, long and short axes, taken across the plasma membrane at its widest point. To ensure measurement accuracy, only sections in which the oocyte profile exhibited maximal cross-sectional area were selected; such sections were identified by systematic examination of serial sections. Follicle diameter was defined as the maximum distance between the outermost boundaries of the granulosa cell layer, likewise measured in the section displaying the largest follicle cross-section.

### 2.3 Transcriptome sequencing

The left ovaries of three striped hamsters exposed to long and short photoperiods were respectively collected for transcriptome sequencing to analyze the effects of photoperiod on ovarian gene expression. The transcriptome sequencing was performed in collaboration with Shanghai OE Biotech Co., Ltd. The specific procedure begins with the extraction of total RNA followed by quality assessment. Subsequently, transcriptome sequencing is performed through a series of steps including mRNA enrichment, RNA fragmentation, reverse transcription, end repair, PCR amplification, and sequencing. Gene count normalization, calculation of fold change, and statistical significance testing are carried out using DESeq2 software. Differentially expressed protein-coding genes and enriched KEGG pathways are then identified based on the fold change (FC) and significance test results. Finally, the resulting data are analyzed using the company’s cloud-based platform.

### 2.4 Determination of reproductive hormones and cAMP concentration

After euthanasia of the striped hamsters, blood samples were collected. The blood was allowed to stand under low-temperature conditions to facilitate stratification, followed by centrifugation at 4°C and 3,000 rpm for 15 minutes using a high-speed refrigerated centrifuge. The resulting pale yellow supernatant was carefully transferred into new EP tubes and stored at -80°C until further analysis. Unilateral ovaries from three female striped hamsters in each group were collected. The tissues were homogenized following lysis to prepare tissue homogenates, and the supernatants were obtained after centrifugation. The cAMP concentrations in both ovarian and serum samples were measured using a hamster-specific cAMP ELISA kit (Item Number: HS011-Hr; Manufacturer: Shanghai Hengyuan Biotechnology Co., Ltd.). Serum levels of follicle-stimulating hormone (FSH) and luteinizing hormone (LH) were quantified using corresponding hamster-specific FSH (Item Number: HS032-Hr; Manufacturer: Shanghai Hengyuan Biotechnology Co., Ltd.) and LH (Item Number: HS034-Hr; Manufacturer: Shanghai Hengyuan Biotechnology Co., Ltd.)ELISA kits, respectively.

### 2.5 Real-time quantitative PCR to assess the mRNA levels

#### 2.5.1 Total RNA extraction from ovarian tissues

Unilateral ovaries from four striped hamsters per group were retrieved from a -80°C ultra-low temperature freezer and transferred into 1.5 mL centrifuge tubes. RNAex lysis buffer (Cat. #9108, Takara) 400 μL was added, and samples were thoroughly homogenized using grinding tools. Following ice-cold incubation, lysates were centrifuged at 12,000 × g for 5 min at 4 °C. The supernatant was transferred to a new RNase-free tube. Chloroform (20% v/v of the supernatant volume) was added, and the mixture was vortexed vigorously for ≥30 s to yield a homogeneous emulsion. After a further 5 min incubation on ice, samples were centrifuged at 12,000 × g for 15 min at 4 °C. The upper aqueous phase was aspirated and transferred to RNase-free tube. An equal volume of ice-cold isopropanol was added, and the solution was gently but thoroughly mixed by inversion. Samples were then incubated at 4 °C for 10 min to ensure RNA precipitation. Subsequent centrifugation at 12,000 × g for 10 min at 4 °C pelleted the RNA; the supernatant was completely removed. The RNA pellet was washed once with 800 μL of ice-cold 75% ethanol (v/v, prepared in DEPC-treated water), followed by centrifugation at 12,000 × g for 5 min at 4 °C; the ethanol supernatant was discarded. The pellet was air-dried briefly, and resuspended in 30 μL of RNase-free water containing 0.1% diethyl pyrocarbonate (DEPC).

RNA purity was assessed spectrophotometrically (Eppendorf, Germany) using 1 μL RNA. Samples with an A260/A280 ratio between 1.8 and 2.0 were considered free of protein and residual phenol contamination. RNA concentration was calculated from the A260 value measured on the same spectrophotometer: concentration (μg/mL) = A260 × dilution factor × 40 μg/mL, where 40 is the standard extinction coefficient for RNA. RNA integrity was evaluated by denaturing agarose gel electrophoresis (1.5% w/v agarose in 1× TAE buffer supplemented with 2.2 M formaldehyde). For electrophoresis, 3 μL of RNA was mixed with 0.5 μL of 6× RNA loading dye, and electrophoresis was performed at 100 V for 30 min in 1× TAE buffer at room temperature. Gels were visualized under UV light using a Gel Doc XR+ system (Bio-Rad, USA). Intact RNA was confirmed by the presence of sharp, well-resolved 28S and 18S ribosomal RNA bands, with the 28S band exhibiting approximately twice the intensity of the 18S band, as well as a distinct 5S rRNA band. The absence of low-molecular-weight smearing or a diffuse background on the electrophoretic gel further confirmed the high integrity of the RNA.

#### 2.5.2 cDNA synthesis

cDNA synthesis was performed using 500 ng of total RNA, with the volume adjusted based on the measured RNA concentration. A 10μL reaction mixture was prepared by adding 2μL of 5×*Evo M-MLV* RT Premix for qPCR (Accurate Biotechnology (Hunan) Co., Ltd) and nuclease-free water to reach the final volume. Reverse transcription was carried out at 37°C for 15 min, followed by 85°C for 5 sec, and then held at 4°C. The resulting cDNA was stored at -80°C for subsequent use.

#### 2.5.3 Primer design for real-time quantitative PCR

The coding sequences (CDS) of key genes in the cAMP signaling pathway—*GPR3*, *GPR12*, *ADCY3*, *ADCY5*, *PDE1A*, and *PDE3A*—were retrieved from NCBI Nucleotide for closely related rodent species. Gene-specific accessions include: *GPR3* (*Cricetulus griseus*: XM_027399555.2; *Phodopus roborovskii*: XM_051188320.1); *GPR12* (*Cricetulus griseus*: XM_007652208.3; *Mesocricetus auratus*: XM_013122814.3); *ADCY3* (*Sciurus vulgaris*: XM_079109625.1; *Meriones unguiculatus*: XM_021648273.2); *ADCY5* (*Cricetulus griseus*: XM_027412774.2; *Microtus oregoni*: XM_041672832.1); *PDE1A* (*Mesocricetus auratus*: XM_013121164.2; *Myodes glareolus*: XM_048425917.1; *Grammomys surdaster*: XM_028762872.1); and *PDE3A* (*Cricetulus griseus*: XM_027437116.2; *Rattus norvegicus*: NM_017337.1; *Phodopus roborovskii*: XM_051173428.1; *Mus musculus*: NM_018779.2). Sequence homology was analyzed using DNAMAN software to identify highly conserved regions, based on which primers for real-time quantitative PCR were designed. The designed primers were synthesized by Shanghai Bioengineering Co., Ltd. Primers and their melting temperatures (Tm) for real-time quantitative PCR of cAMP pathway-related genes are listed in **Table 1**.

**Table 1:** Primers for real-time quantitative PCR of cAMP pathway-related genes.

Primer	Primer sequences	Product length (bp)	Annealing temperature (°C)
GPR3	F: 5′-TGCTGGTTGCCCTTCACTGTC-3′	131	64
	R: 5′-TGGTTGCGGAAGGCGTAGATG-3′		
GPR12	F: 5′-GGCTGGAACTGCTTGAGGGATG-3′	91	66
	R: 5′-AGAGGAAGGAGATGGACAGGATGG-3′		
ADCY3	F: 5′-GGTGGAGGCCATCTCGTATG-3′	165	61
	R: 5′-CCACCAGCCTCCATCTTG-3′		
ADCY5	F: 5′-TTGGTGGTGTTTGTGTCCGT-3′	107	60
	R: 5′-ACCAGGGTGCTGTTTGTCTT-3′		
PDE1A	F: 5′-AACTTGGGAGCTGCACATCA-3′	137	60
	R: 5′-TTTGAGACTGGGCAACCATC-3′		
PDE3A	F: 5′-TGCATGACTACGATCACCCG-3′	139	60
	R: 5′-GGCCGGGACATGAAGAGATT-3′		
β-actin	F: 5′-GAGACCTTCAACACCCCAGC-3′	256	n
	R: 5′-ATGTCACGCACGATTTCCC-3′		

#### 2.5.4 Real-time quantitative PCR

In this study, β-actin was used as the internal reference gene, and gene expression levels were quantified by real-time quantitative PCR using the BIO-RAD CFX Connect™ system. The 20 μL reaction mixture contained 0.5 μL of forward primer and 0.5 μL of reverse primer, 10 μL of 2× SYBR Green Pro Taq HS Premix, 2 μL of cDNA template, and nuclease-free water to adjust the final volume to 20 μL. The amplification protocol consisted of an initial denaturation at 95 °C for 30 s, followed by 40 cycles of denaturation at 95 °C for 5 s and annealing at Tm °C (as specified in **Table 1**) for 30 s. The resulting Ct values were analyzed using the 2^–ΔΔCt^ method to determine the relative expression levels of the target genes.

### 2.6 Data processing and statistical analysis

All data obtained in this study are presented as mean ± standard error of the mean (SEM). One-way analysis of variance (ANOVA) was performed using IBM SPSS Statistics 26.0 to assess differences among groups, with statistical significance defined as *P* < 0.05, high significance as *P* < 0.01, and non-significance as *P* > 0.05. Bar graphs illustrating group comparisons were generated using GraphPad Prism 8. Correlation analyses were conducted using GraphPad Prism 8 based on individual data points from experimental animals to evaluate relationships between variables.

## 3 Results

### 3.1 Differences in body weight, ovary weight, and ovarian coefficient in striped hamsters under different photoperiods

**Figure 1:**
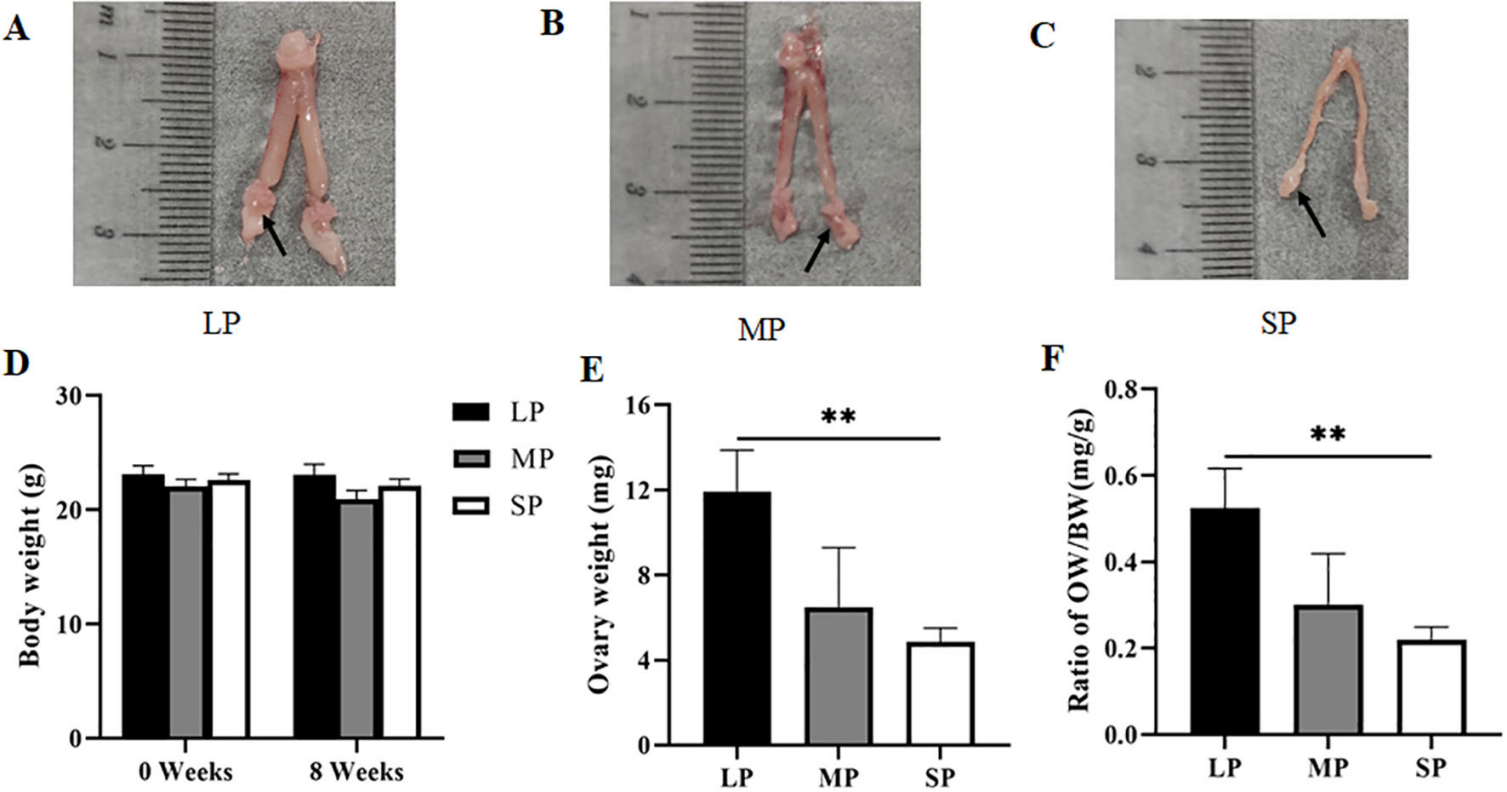
Differences in body weight, ovary weight, and ovarian coefficient in striped hamsters under different photoperiods. **(A)** Reproductive organs of the LP group, arrow indicating the ovary; **(B)** Reproductive organs of the MP group, arrow indicating the ovary; **(C)** Reproductive organs of the SP group, arrow indicating the ovary; **(D)** Body weight; **(E)** Unilateral ovary weights; **(F)** Unilateral ovarian coefficient. LP, long photoperiod, n = 13; MP, medium photoperiod, n = 5; SP, short photoperiod, n = 14; values are the mean ± standard error of the mean. *P < 0.05 or **P < 0.01 represents significant difference.

In this study, adult female striped hamsters (5 months of age) were subjected to different photoperiod treatments for 8 weeks. Body weights were measured before and after treatment, and the results are presented in **Figure 1D**. No significant differences in body weight were observed among the groups before or after photoperiod exposure (*P* > 0.05), indicating that photoperiod does not exert a significant effect on body weight gain in adult female striped hamsters. Following completion of the photoperiod treatment, animals were euthanized and dissected. **Figures 1A–C** illustrate the reproductive organs of female striped hamsters, including the uterus, oviducts, ovaries (indicated by black arrows), and the periovarian adipose tissue. After 8 weeks of photoperiod treatment, striped hamsters in the long photoperiod group (LP) exhibited a thicker and more robust uterus and larger ovaries; the medium photoperiod group (MP) showed intermediate morphological features, whereas those in the short photoperiod group (SP) had a slender uterus and smaller ovaries. Following dissection of the oviducts and periovarian adipose tissue, the left ovary was excised, weighed, and used to calculate the ovarian coefficient. Results are presented in **Figures 1E, F**. Unilateral ovarian weight and ovarian coefficient were significantly higher in the LP group than in the SP group (*P* < 0.01). No significant differences were observed between the MP group and either the LP or SP groups (*P* > 0.05); however, both unilateral ovarian weight and ovarian coefficient in the MP group showed a tendency to increase relative to the SP group. These findings indicate that an 8-week photoperiod significantly modulates ovarian development in adult female striped hamsters, while exerting no detectable effect on body weight under standardized feeding conditions (2–3 g of standard laboratory rodent chow per animal per day). Long photoperiods may promote ovarian development, whereas short photoperiods may suppress it, ultimately resulting in ovarian atrophy and degeneration.

### 3.2 Differences in ovarian morphological structure and the number of follicles at various developmental stages in striped hamsters under different photoperiods

**Figure 2:**
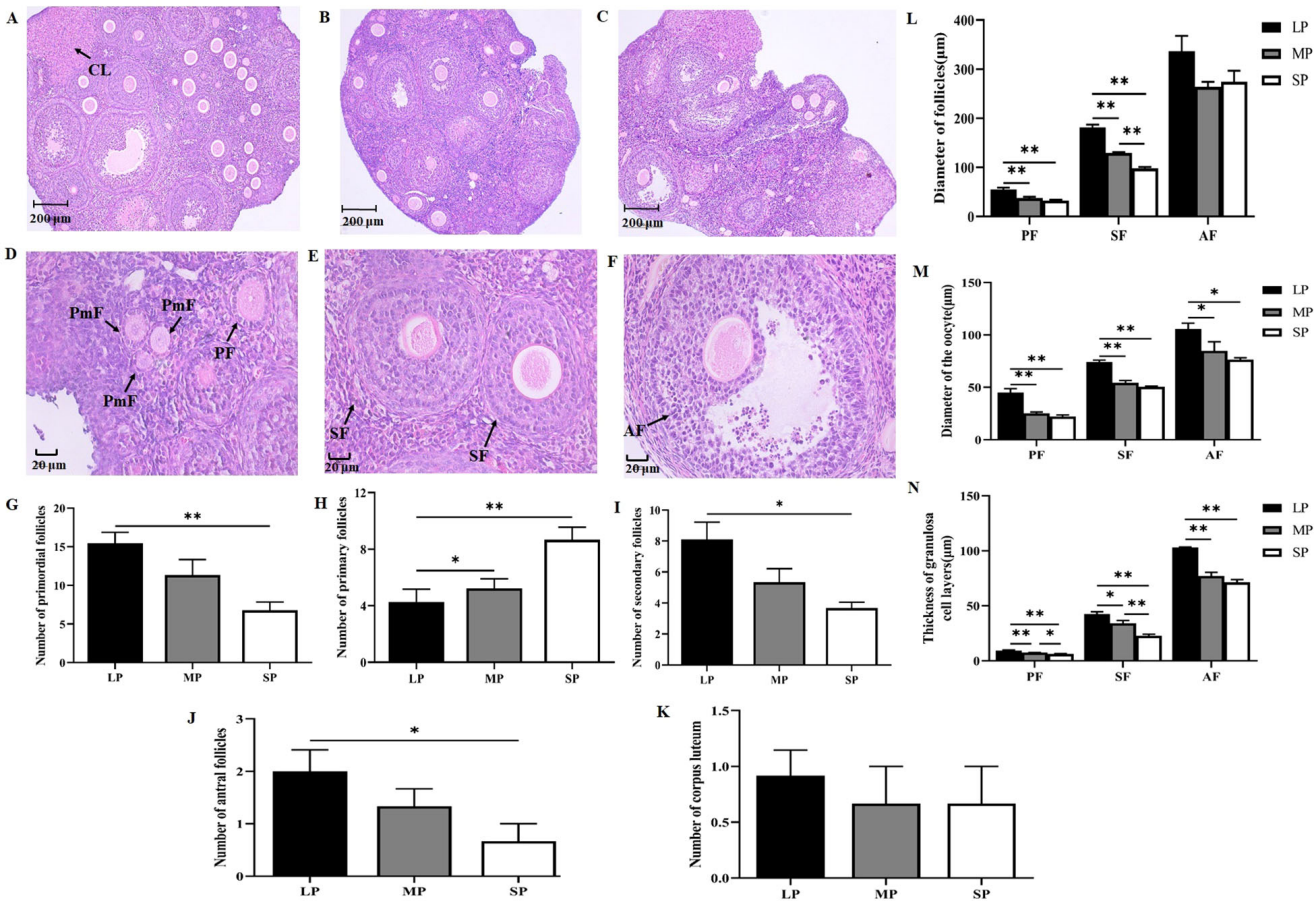
Differences in HE staining, follicle numbers at equivalent developmental stages, and follicle architecture in striped hamsters exposed to different photoperiods. **(A-C)** HE staining of ovarian sections from LP **(A)** MP **(B)** and SP **(C)** groups at 10×magnification; **(D-F)** HE staining of follicles at various developmental stages at 40×magnification; **(G-K)** Comparison of primordial follicle **(G)** primary follicle **(H)** secondary follicle **(I)** antral follicle **(J)** and corpus luteum **(K)** counts across photoperiod groups; **(L)** Follicle diameter differences among groups; **(M)** Oocyte diameter differences among groups; **(N)** Granulosa cell layer thickness differences among groups. LP, long photoperiod; MP, medium photoperiod; SP, short photoperiod; PmF, primordial follicle; PF, primary follicle; SF, secondary follicle; AF, antral follicle; CL, corpus luteum. n = 3; values are expressed as mean ± standard error of the mean (SEM). *P < 0.05 or **P < 0.01 indicates statistically significant differences.

**Figures 2A–C** present HE-stained sections of the maximal cross-sectional area of ovaries from LP, MP, and SP groups, respectively. Significant differences exist in the morphological structure of follicles across developmental stages. Primordial follicles consist of a single layer of flattened granulosa cells enclosing the oocyte and exhibit the smallest follicular diameter (**Figure 2D**). Primary follicles are larger than primordial follicles, maintaining a single layer of granulosa cells, but the cells transition from flattened to cuboidal in shape (**Figure 2D**). Secondary follicles are further enlarged, with granulosa cells undergoing proliferation to form multiple layers of tightly packed cells; a well-defined zona pellucida surrounding the oocyte is clearly visible (**Figure 2E**). Antral follicles develop a fluid-filled antral cavity within the follicle, accompanied by an increased number of granulosa cell layers, although the cellular arrangement becomes less compact (**Figure 2F**). The corpus luteum exhibits lighter staining intensity and greater overall volume (**Figure 2A**).

Significant differences were observed in the distribution of follicle numbers across developmental stages in the ovaries of female striped hamsters under different photoperiods. As shown in **Figure 2G**, the number of primordial follicles increased with prolonged light exposure duration, with the LP group exhibiting an extremely significant increase compared to the SP group (*P* < 0.01). Primary follicle counts are presented in **Figure 2F**. Primordial follicles can be selectively activated and differentiate into primary follicles. At this developmental stage, the LP group had significantly fewer primary follicles than the MP group (*P* < 0.05) and markedly fewer than the SP group (*P* < 0.01). Further analysis revealed that the numbers of secondary and antral follicles were significantly higher in the LP group compared to the SP group (**Figures 2I, J**, *P* < 0.05), whereas no significant differences were observed in luteal cell counts across groups (**Figures 2K**, *p* > 0.05). These findings suggest that long photoperiods may promote the progression of primary follicles into secondary and antral follicles, thereby supporting follicle development.

The diameters of primary, secondary, and antral follicles, oocyte diameters, and granulosa cell layer thickness in the ovaries of female striped hamsters under different photoperiods were analyzed. Results are presented in **Figure 2L**. Primary and secondary follicle diameters in the LP group were highly significantly greater than those in both the MP and SP groups (*P* < 0.01). Additionally, secondary follicle diameter in the MP group was significantly larger than in the SP group (*P* < 0.01), whereas no significant differences were observed in antral follicle diameter among the three groups (*P* > 0.05). Oocyte diameter across different developmental stages was significantly greater in the LP group compared to both the MP and SP groups (**Figure 2M**, *P* < 0.05). The granulosa cell layer thickness in primary, secondary, and antral follicles was also markedly increased in the LP group relative to the MP and SP groups (**Figure 2N**, *P* < 0.05). Furthermore, within the MP group, granulosa cell layer thickness in primary follicles was significantly higher than in the SP group (*P* < 0.05), and this difference was even more pronounced in secondary follicles, showing an extremely significant increase (*P* < 0.01). These findings demonstrate that photoperiod regulates the developmental progression of follicles at various preovulatory stages. Long photoperiods enhance granulosa cell proliferation and oocyte maturation, thereby facilitating follicle development.

### 3.3 Transcriptome sequencing analysis of ovarian tissues from striped hamsters under different photoperiods

**Figure 3:**
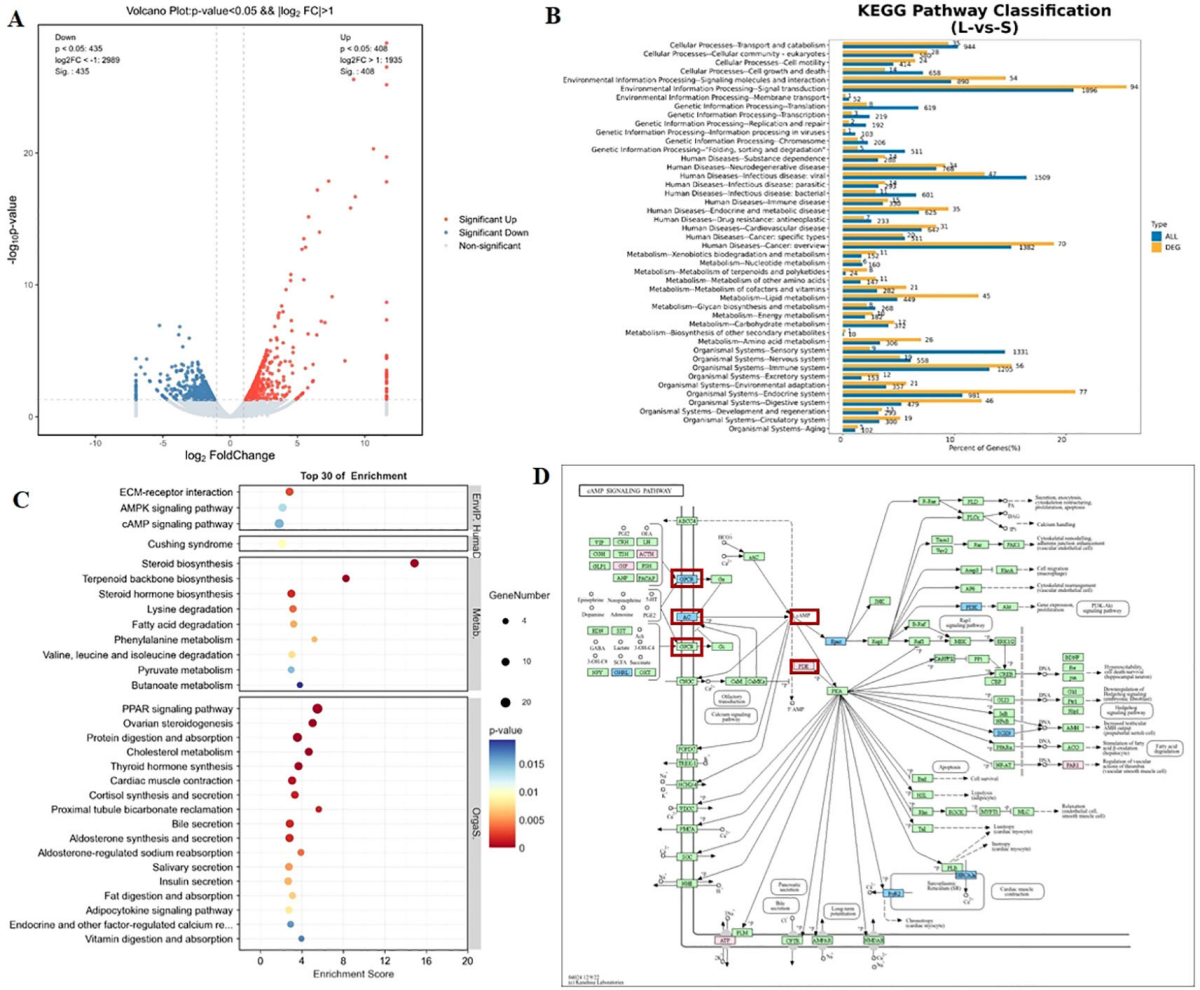
Transcriptome analysis of ovarian tissues from striped hamsters under varying photoperiods. **(A)** Volcano plot of differentially expressed genes in ovarian tissues under varying photoperiods. Sig denotes the number of significantly differentially expressed genes; the horizontal axis represents log2 fold change, and the vertical axis represents -log10 (p-value). **(B)** Comparison of genes mapped to different Level-2 KEGG pathways. The vertical axis shows the pathway names, the horizontal axis represents the percentage of genes, and the number to the right of each bar indicates the number of genes assigned to the corresponding pathway entry. **(C)** Bubble plot of KEGG enrichment analysis. The vertical axis represents the KEGG pathway name, with level 1 categories on the right and level 3 sub-pathways on the left; the horizontal axis shows the enrichment score. **(D)** cAMP signaling pathway. Genes highlighted in red boxes represent differentially expressed genes within the cAMP signaling pathway under varying photoperiods.

To investigate the molecular mechanisms underlying ovarian follicle development in female striped hamsters in response to photoperiodic changes, transcriptome sequencing was conducted on ovarian tissues from individuals in the LP (n = 3) and SP (n = 3) groups, aiming to identify differentially expressed genes and elucidate their associated functional pathways. As shown in **Figure 3**, a total of 843 differentially expressed genes were identified between the two groups. Compared with the SP group, 408 genes were significantly upregulated and 435 genes were significantly downregulated in the LP group (**Figure 3A**). KEGG pathway enrichment analysis of differentially expressed genes in the ovarian tissues from the two groups revealed that the Signal transduction pathway at level 2 contained both the highest number and the greatest proportion of differentially expressed genes, and it belongs to the Environmental Information Processing category at level 1 (**Figure 3B**). Further analysis of the level 3 pathways within this category revealed that the cAMP signaling pathway, AMPK signaling pathway, and calcium signaling pathway exhibited the lowest P-values, whereas the PI3K-Akt signaling pathway, cAMP signaling pathway, and calcium signaling pathway had the highest numbers of differentially expressed genes, ranking among the top three. A bubble plot of the top 30 KEGG enriched pathways at level 3 (**Figure 3C**) revealed significant enrichment of genes in the cAMP signaling pathway and other related pathways, suggesting a potential role of the cAMP signaling pathway in photoperiod-regulated ovarian development in striped hamsters.

Differentially expressed genes in the cAMP signaling pathway with *P* < 0.05 and fold change (FC) > 2 were selected and highlighted in red (**Figure 3D**). Compared with the SP group, the LP group exhibited downregulated expression of the cAMP synthase AC-related gene *ADCY5* and *GPCRs* involved in regulating ADCY synthesis. The mRNA expression of the cAMP-degrading enzyme-associated gene *PDE1A* was significantly upregulated, along with marked downregulation of genes encoding downstream cAMP effector proteins, including *Rapgef3* and *Pik3cd*. The above results suggest that photoperiod regulates the expression of genes associated with the cAMP signaling pathway, with significantly differentially expressed genes predominantly involved in the processes of cAMP synthesis and degradation.

### 3.4 Differences in gonadotropin levels and cAMP concentrations in striped hamsters under different photoperiods

**Figure 4:**
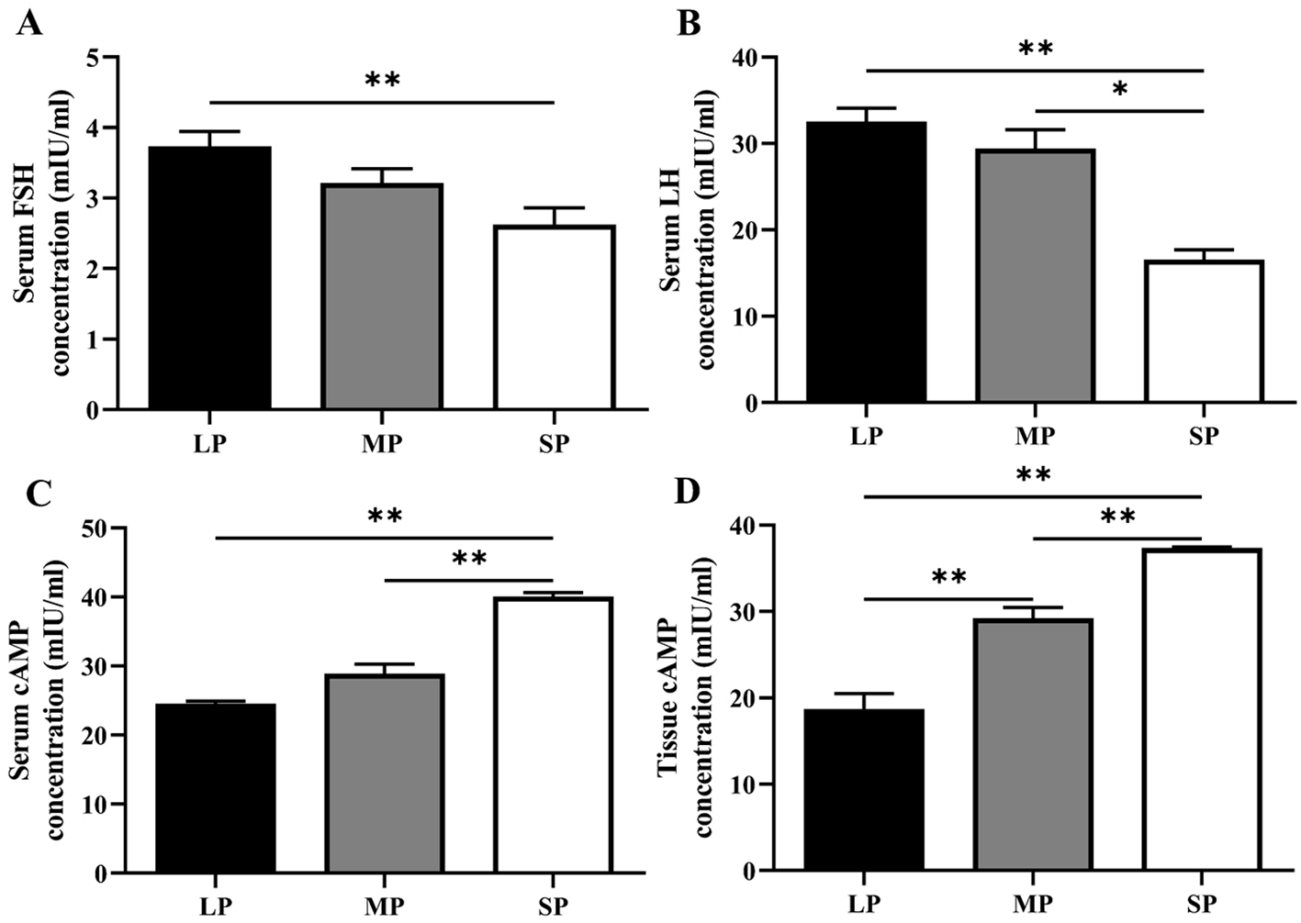
Differences in serum gonadotropin and cAMP concentrations, as well as ovarian cAMP content, in striped hamsters under different photoperiods. **(A-C).** Serum FSH **(A)**, serum LH **(B)**, and serum cAMP **(C)** levels. LP: long photoperiod, n = 13; MP: medium photoperiod, n = 5; SP, short photoperiod, n = 14. **(D)** Ovarian tissue cAMP content. LP: n = 3; MP: n = 3; SP: n = 3. Values are presented as mean ± standard error of the mean (SEM). *P < 0.05 and **P < 0.01 indicate statistically significant differences.

Gonadotropins regulate the synthesis and release of sex hormones, which in turn modulate follicle development. Significant differences were observed in serum FSH and LH concentrations across different photoperiods (**Figures 4A, B**). Serum FSH and LH levels in female striped hamsters exposed to long photoperiods (LP) were markedly higher than those in animals under short photoperiods (SP) (*P* < 0.01). Moreover, LH concentration in the MP group was significantly elevated compared to the SP group (*P* < 0.05). The results shown in **Figures 4C, D** demonstrate that cAMP levels in both serum and ovarian tissue were significantly higher in the SP group than in the MP and LP groups (*P* < 0.01). Furthermore, cAMP concentration in the MP group was significantly elevated compared to the LP group (*P* < 0.01). The observed differences in FSH, LH, and cAMP concentrations indicate that photoperiod modulates the synthesis and secretion of these hormones in female striped hamsters. Long photoperiod (LP) promotes gonadotropin production and release but suppresses cAMP synthesis and secretion. Notably, cAMP content in ovarian tissue exhibits a marked increase as light duration decreases. Thus, photoperiod regulates follicle development through coordinated control of FSH and LH secretion and cAMP concentration.

### 3.5 The correlation among gonadotropin levels, cAMP levels, and follicle number

**Figure 5:**
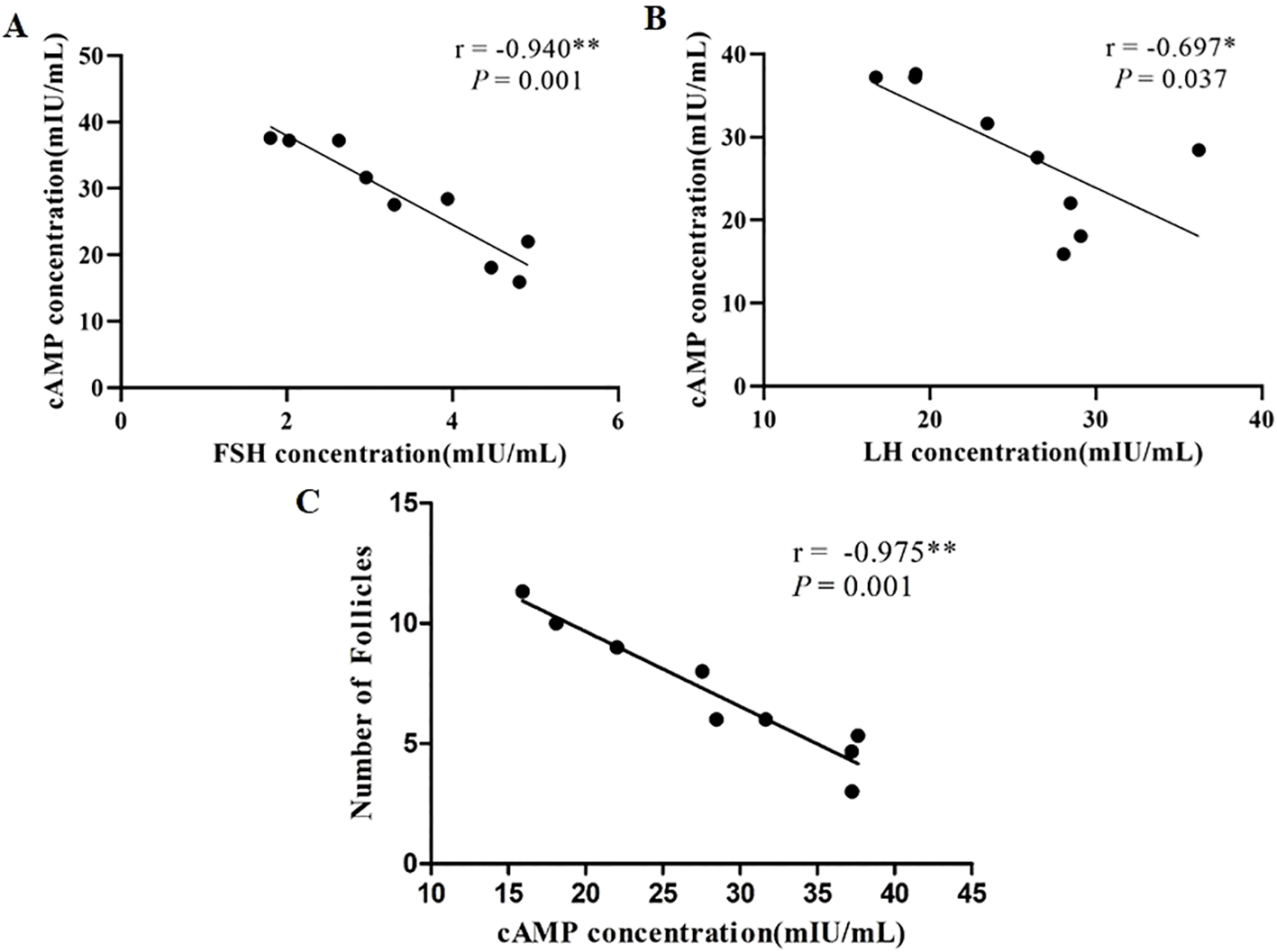
Correlation between serum gonadotropin levels and ovarian cAMP levels, and between ovarian cAMP levels and follicle numbers. **(A)** correlation between FSH and cAMP; **(B)** correlation between LH and cAMP; **(C)** correlation between cAMP levels and follicle numbers; n = 9; r denotes the correlation coefficient; *P < 0.05 and **P < 0.01 indicate statistically significant differences.

To investigate the regulatory effects of gonadotropins on cAMP synthesis, this study examined the correlations between serum FSH and LH levels and ovarian cAMP levels in female striped hamsters. The results are presented in **Figures 5A, B**. Serum FSH levels showed a highly significant negative correlation with ovarian cAMP levels (r = -0.940, *P* < 0.01), while serum LH levels exhibited a significant negative correlation (r = -0.697, *P* < 0.05). These findings suggest that ovarian cAMP levels are negatively regulated by both FSH and LH, with FSH exerting a stronger regulatory influence than LH.

To investigate the regulatory role of cAMP in follicle development in female striped hamsters, this study examined the correlation between ovarian cAMP levels and the total number of secondary and antral follicles. The results are presented in **Figure 5C**. Ovarian cAMP levels showed a highly significant negative correlation with follicle number (r = -0.957, *P* < 0.01). These findings indicate that elevated ovarian cAMP levels are associated with impaired development of secondary and antral follicles. Furthermore, the increased cAMP levels observed in the ovaries of female striped hamsters under short photoperiods may suppress folliculogenesis via specific downstream signaling pathways.

### 3.6 Differences in the relative mRNA expression levels of cAMP pathway-related factors under different photoperiods

**Figure 6:**
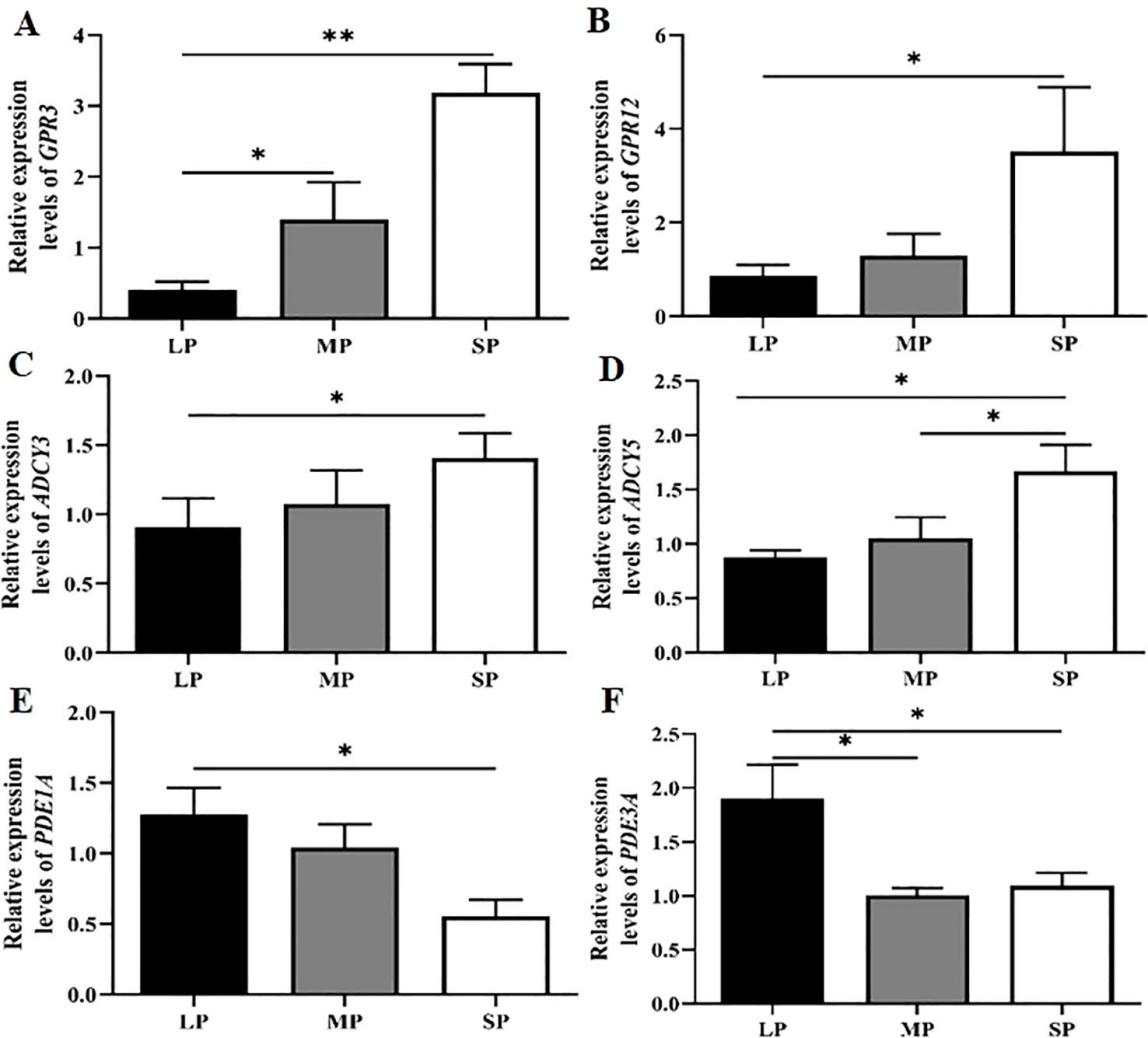
Differences in the relative mRNA expression of genes related to the cAMP signaling pathway under varying photoperiods. **(A-F)** Relative mRNA expression levels of GPR3 **(A)** GPR12 **(B)** ADCY3 **(C)** ADCY5 **(D)** PDE1A **(E)** and PDE3A **(F)** under different photoperiods; n = 4; data are presented as means ± standard deviation. *P < 0.05 and **P < 0.01 indicate statistically significant differences.

This study utilized real-time quantitative PCR to examine the differences in relative mRNA expression levels of key regulatory factors involved in cAMP homeostasis within the cAMP signaling pathway, specifically *ADCY3*, *ADCY5*, *PDE1A*, *PDE3A*, *GPR3*, and *GPR12*, in the ovaries of female striped hamsters exposed to different photoperiods. As shown in **Figures 6A, B**, the relative mRNA expression levels of *GPR3* and *GPR12*, upstream regulators of cAMP synthesis, were highest in the short photoperiod (SP) group. Under long photoperiods (LP), *GPR3* mRNA expression was significantly lower than in the medium photoperiod (MP) group (*P* < 0.05) and markedly reduced compared to the SP group (*P* < 0.01). Similarly, *GPR12* mRNA expression in the LP group was significantly lower than in the SP group (*P* < 0.05). As shown in **Figures 6C, D**, the relative mRNA expression levels of *ADCY3* and *ADCY5*—key enzymes involved in cAMP synthesis—were significantly lower in the long photoperiod (LP) group compared to the short photoperiod (SP) group. Moreover, *ADCY5* expression in the medium photoperiod (MP) group was significantly reduced relative to the SP group (*P* < 0.05), suggesting that shorter photoperiods promote the expression of cAMP-synthesizing enzymes. The relative mRNA expression levels of *PDE1A* and *PDE3A*, key enzymes involved in cAMP degradation, were significantly higher under long photoperiods compared to short photoperiods. Furthermore, *PDE3A* expression in the MP group was significantly elevated relative to the SP group (**Figures 6E, F**, *P* < 0.05). The expression pattern of the cAMP-degrading enzymes was opposite to that of the synthesizing enzymes. These findings indicate that cAMP-synthesizing and cAMP-degrading enzymes may function coordinately to reduce ovarian cAMP concentrations in female striped hamsters exposed to long photoperiods. The results demonstrate that photoperiod modulates the mRNA expression levels of cAMP-related genes, including the upstream regulatory factors GPR3 and GPR12, the synthesizing enzymes ADCY3 and ADCY5, and the degrading enzymes PDE1A and PDE3A. Long photoperiods promote the expression of cAMP-degrading enzyme genes while suppressing the expression of upstream regulators and synthesizing enzymes. Thus, long photoperiods may reduce ovarian cAMP levels in female striped hamsters by simultaneously inhibiting cAMP synthesis and enhancing its degradation, thereby fine-tuning cAMP concentration in the ovary. As a key signaling molecule, cAMP subsequently regulates follicle development in female striped hamsters.

### 3.7 Correlation analysis between the expression levels of key components in the cAMP signaling pathway and ovarian cAMP content

The intracellular cAMP level is coordinately regulated by membrane-bound G protein-coupled receptors, adenylyl cyclases responsible for cAMP synthesis, and phosphodiesterases involved in cAMP degradation. This study investigated the correlation between ovarian cAMP levels and the mRNA expression of key regulatory genes, *GPR3*, *GPR12*, *ADCY3*, *ADCY5*, *PDE1A*, and *PDE3A*, and further explored their potential roles in modulating cAMP content in the ovaries of striped hamsters. As shown in **Table 2**, the mRNA expression levels of *GPR3* and *ADCY5* were highly significantly and significantly positively correlated with cAMP levels in the ovaries of striped hamsters (*P* < 0.01 and *P* < 0.05, respectively), while those of *PDE1A* and *PDE3A* exhibited highly significant and significant negative correlations with ovarian cAMP content (*P* < 0.01 and *P* < 0.05, respectively). In contrast, no significant associations were observed between cAMP levels and the mRNA expression of *ADCY3* or *GPR12* (*P* > 0.05). The research findings indicate that GPR3, an upstream regulator of cAMP synthesis, together with the cAMP-synthesizing enzyme ADCY5 and the cAMP-degrading enzymes PDE1A and PDE3A, plays critical roles in mediating photoperiodic regulation of ovarian cAMP levels in striped hamsters. These genes respond to photoperiods and coordinately regulate ovarian cAMP content, thereby modulating follicle development in striped hamsters.

**Table 2:** Correlation between the expression levels of cAMP-related factors and cAMP content. .

cAMP	mRNA levels
GPR3	r = 0.8556**, *P* = 0.0033
GPR12	r = 0.6083, *P* = 0.0822
ADCY3	r = 0.4272, *P* = 0.2515
ADCY5	r = 0.6729*, *P* = 0.0470
PDE1A	r = -0.8026**, *P* = 0.0092
PDE3A	r = -0.7014*, *P* = 0.0352

n = 9; *P < 0.05 and **P < 0.01 indicate significant differences.

## 4 Discussion

Temperate regions are characterized by pronounced seasonal climatic fluctuations, with marked differences among seasons. Seasonal breeding mechanisms in response to photoperiod vary among species. The neuroendocrine basis of long-day seasonal breeding in striped hamster remains poorly understood. In this study, adult female striped hamsters were exposed to LP, MP, and SP conditions for 8 weeks to investigate the underlying mechanisms of photoperiod-regulated seasonal reproduction in this species.

### 4.1 Effects of photoperiod on body weight, ovarian weight, and ovarian coefficient in female striped hamsters

The striped hamster is a photoperiod-sensitive seasonal breeder. In this study, no significant effect of photoperiod on body weight was observed in 5-month-old adult females, a finding consistent with that reported by Xue et al. (11) in 8-month-old animals, but inconsistent with the results of Wang et al. (38). This discrepancy may stem from differences in experimental duration and/or dietary intake. Specifically, Wang et al. (38) exposed adult female striped hamsters to controlled photoperiod conditions for 10 weeks and provided about 4–5 g of standard laboratory rodent chow per animal per day, exceeding typical metabolic requirements for most individuals. In contrast, our study employed an 8-week photoperiod exposure and supplied 2–3 g of the same chow daily, which aligns closely with established maintenance requirements for adult striped hamsters under standard housing conditions. In contrast, short photoperiods have been shown to induce substantial body weight loss in Siberian hamsters (39), highlighting a species-specific divergence in photoperiodic regulation of energy balance. This discrepancy suggests that the regulatory influence of photoperiod on body weight in seasonal breeders depends on species, the duration and pattern of photoperiod exposure, and dietary intake. Photoperiod significantly influences ovary weight and ovarian coefficient in 5-month-old female striped hamsters, consistent with findings reported in 8-month-old adult females (11). The LP group exhibited markedly higher ovary weight and ovarian coefficient compared to the SP group, a finding that aligns with previous studies in golden hamsters (40). The above analysis indicates that photoperiod does not significantly influence body weight in female striped hamsters, yet it exerts a significant regulatory effect on ovarian developmental status. Shorter light duration was associated with a decreasing trend in ovary weight, and ovarian development was impaired compared to long-day conditions, characterized by hypoplastic and atrophic changes. The physiological activities of female mammals are closely linked to the functional status of their organs. As the primary gonad, the ovary plays a critical role in regulating reproductive processes, and impaired ovarian development is associated with reduced reproductive capacity. In this study, ovarian development in female striped hamsters was markedly better under LP conditions than under SP conditions. These findings indicate that LP promotes reproductive activity, whereas SP suppress gonadal development and induce a non-reproductive state in female striped hamsters.

### 4.2 The effect of photoperiod on follicle development in female striped hamsters

Histological observations revealed significant differences in the number and developmental status of follicles across various stages between photoperiod groups. Specifically, the LP group exhibited significantly higher numbers of secondary and antral follicles but significantly lower numbers of primary follicles than the SP group, a finding consistent with that reported by Xue et al. (11). Studies have demonstrated that SP impairs follicle development in Siberian hamsters, leading to decreased follicle numbers and exacerbated follicle atresia; in contrast, LP exposure restores ovarian function (41, 42). In addition, no significant difference was observed in corpus luteum number among photoperiod groups, a finding that contrasts with report (11) of significantly higher corpus luteum numbers in the LP group compared with the SP group. Although corpus luteum number tended to increase with longer photoperiod, this trend did not reach statistical significance. This discrepancy may be attributable to age differences between the experimental cohorts, suggesting that 8-month-old female striped hamsters exhibit higher reproductive competence than 5-month-old individuals. Therefore, it is hypothesized that LP promotes follicle development in female striped hamsters, resulting in a significantly greater number of secondary and antral follicles in the LP group compared to the SP group. In contrast, SP induces follicle atresia, with most follicles failing to reach the ovulatory stage, thereby reducing the numbers of secondary and antral follicles in the SP group. Therefore, LP may help preserve the primordial follicle reserve, promote oocyte growth and granulosa cell proliferation, and reduce follicle atresia in female striped hamsters, whereas short photoperiod exerts opposing effects.

### 4.3 Transcriptome analysis reveals the molecular pathways underlying ovarian responses to photoperiod to regulate follicle development in striped hamsters

Comprehensive transcriptome analysis of ovarian tissues from animals exposed to LP or SP revealed significant enrichment of differentially expressed genes in the cAMP signaling pathway, with a highly statistically significant *P* value. Analysis of differentially expressed genes in the cAMP signaling pathway revealed that these genes were primarily upstream regulators of cAMP synthase expression, cAMP synthase itself, and cAMP-degrading enzymes. This indicates that photoperiod regulates ovarian cAMP levels by modulating the rates of cAMP synthesis and degradation, thereby influencing follicle development. Evidence from multiple studies indicates that cAMP regulates ovarian development via diverse signal transduction pathways (21). Elevated cAMP levels can accelerate primordial follicle activation (43), maintain the arrest of the first meiotic division in primary oocytes—thereby inhibiting oocyte maturation (44)—and regulate granulosa cell proliferation (45). These findings are consistent with the results of the present study, which show that female striped hamsters in the LP group exhibited lower ovarian cAMP levels, a condition favorable for oocyte growth and granulosa cell proliferation, thus promoting follicle development. Photoperiod likely exerts its effects primarily by modulating the expression of genes encoding upstream regulators of cAMP, including GPCRs, cAMP synthase, and cAMP-degrading enzymes, thereby altering ovarian cAMP levels and subsequently regulating follicle development.

### 4.4 Photoperiod regulates the synthesis of gonadotropins and cAMP to influence follicle development

Photoperiod regulates the synthesis and secretion of gonadotropins, thereby modulating follicle development (46). Consistent with this mechanism, the present study found that serum FSH and LH concentrations were significantly higher in female striped hamsters exposed to LP than in those under SP; moreover, LH levels in the MP group were also significantly higher relative to the SP group. LP exposure robustly stimulates gonadotropin synthesis and secretion, promoting follicle growth, differentiation, and ovulation (47), whereas SP suppresses FSH and LH production and release in seasonal breeders, leading to delayed follicle development, anovulation, and ultimately reproductive quiescence (48). These findings collectively support the photoperiod-dependent regulation of the hypothalamic-pituitary-gonadal axis in striped hamsters. Previous studies demonstrate that female mice lacking luteinizing hormone (LH) receptors fail to develop antral follicles and exhibit significantly reduced ovarian volume relative to wild-type controls (49). In rats, LH promotes the growth of preantral follicles, and experimentally elevated LH levels can drive their expansion to antral follicles (50). In golden hamsters, serum FSH concentrations are photoperiodically regulated, significantly higher under LP than SP conditions, and FSH facilitates follicle development by stimulating granulosa cell proliferation (40). Collectively, these findings corroborate the central role of gonadotropins in mediating photoperiodic control of follicle development, consistent with the present results. In conclusion, serum FSH and LH concentrations were significantly higher in female striped hamsters exposed to LP than in those under SP. Circulating gonadotropins reach the ovaries via the bloodstream to exert direct regulatory effects on ovaries; elevated FSH and LH levels likely promote follicle development through receptor-mediated signaling ways.

This study demonstrates that serum and ovarian cAMP levels in female striped hamsters are photoperiod-dependent. Specifically, serum and ovarian cAMP levels was significantly lower in LP group than in both MP and SP groups; moreover, the MP group exhibited significantly lower cAMP levels than the SP group. These graded reductions in cAMP across increasingly longer photoperiods indicate an inverse relationship between day length and cAMP levels. Supporting this interpretation, emerging evidence shows that melatonin, whose secretion is tightly gated by photoperiod, regulate follicle development via binding to its MT1 receptor, leading to affecting intracellular cAMP accumulation in sheep oocytes (51). The findings in this study thus align with and extend prior work by demonstrating that endogenous photoperiod-driven melatonin rhythms may orchestrate ovarian cAMP dynamics in a quantitative, duration-dependent manner.

Correlation analysis revealed significant negative associations between serum FSH or LH concentrations and cAMP levels in female striped hamsters, with the FSH-cAMP correlation (r=−0.940, *P* = 0.001) being significantly stronger than the LH-cAMP correlation (r=−0.697, *P*= 0.037). Consistent with these *in vivo* observations, *in vitro* studies demonstrate that exogenous FSH suppresses intracellular cAMP accumulation in rat gonadal cells (18, 52), and LH similarly attenuates cAMP levels in ovarian tissue cells (17). Together, these evidence support a conserved gonadotropin-mediated suppression of ovarian cAMP signaling. We therefore propose that elevated circulating FSH and LH in striped hamsters act synergistically or additively to inhibit cAMP synthesis in ovarian cells, thereby dampening downstream PKA-dependent signaling and modulating follicle development. Critically, the stronger statistical association and greater magnitude of suppression observed with FSH suggest that FSH plays a quantitatively dominant role in photoperiod-regulated cAMP dynamics within the ovary. Elevated cAMP levels, documented in studies, promote excessive activation of primordial follicles (20, 35), thereby disrupting the tightly regulated, stepwise progression of follicle development in female mammals. This mechanism aligns with the observed primordial follicle overactivation and accelerated depletion in the SP group. Given that orderly, spatially restricted, and temporally controlled primordial follicle activation is fundamental to maintaining ovarian reserve and ensuring sustained reproductive capacity, its dysregulation may precipitate premature ovarian insufficiency and compromise fertility. In contrast, prolonged photoperiod exposure appears to preserve the primordial follicle pool, supporting more stable follicle development and extending reproductive lifespan. Conversely, short photoperiod exposure induces granulosa cell apoptosis and suppresses cellular proliferation, leading to a marked increase in atretic follicles (53, 54). Collectively, these findings indicate that cAMP serves as a key intracellular mediator linking photoperiodic cues to the regulation of follicle recruitment and ovarian reserve dynamics.

In this study, the diameters of primary and secondary follicles, oocyte diameter, and granulosa cell layer thickness were significantly greater in LP group than in MP and SP groups. Similarly, oocyte diameter and granulosa cell layer thickness in antral follicles were significantly increased in the LP group relative to both MP and SP groups. These morphological differences suggest that photoperiod exerts regulatory control over granulosa cell proliferation, thereby promoting coordinated follicle growth and maturation. Consistent with this, several studies report that short photoperiod exposure suppresses granulosa cell proliferation, accelerates follicle atresia, and ultimately compromises ovarian function and reproductive performance (41, 55). Notably, supplementation of granulosa cell cultures with 8-Br-cAMP induces apoptosis in over 90% of rat granulosa cells within 15 hours (56), providing direct experimental evidence that elevated intracellular cAMP triggers granulosa cell apoptosis and contributes mechanistically to follicle atresia. This cAMP–apoptosis axis is further supported by our SP-group data, which show concurrently elevated ovarian cAMP levels and reduced follicle counts.

Collectively, these results indicate that photoperiod regulates pituitary secretion of FSH and LH in female striped hamsters, thereby modulating ovarian cAMP signaling. Within this endocrine–intracellular cascade, cAMP acts as a pivotal second messenger that directionally governs follicle dynamics: it regulates follicle development while simultaneously regulating the balance between granulosa cell proliferation and apoptosis, which is essential for maintaining follicle pool homeostasis and developmental competence.

### 4.5 The primary factors mediating photoperiodic regulation of ovarian cAMP levels in female striped hamsters

As a key second messenger, cAMP activates downstream effectors that orchestrate granulosa cell proliferation, steroidogenesis, and oocyte–cumulus communication, thereby coordinating follicle development. This study reveals that ovarian mRNA expression of *GPR3* and *GPR12* in the striped hamster is photoperiod-dependent: both genes are significantly downregulated under LP condition compared with SP (*P* < 0.05). Moreover, GPR3 expression shows a highly significant positive correlation with cAMP concentrations (r=0.856**, *P* = 0.003), whereas no statistically significant association is detected between GPR12 expression and cAMP levels (r=0.608, *P* = 0.082). These findings indicate that GPR3 critically sustains cAMP production in the striped hamster, consistent with evidence from murine oocytes (57) demonstrating its essential role in maintaining meiotic arrest. For LP group, downregulation of GPR3 in the oocyte reduces intra-oocyte cAMP levels, thereby relieving meiotic arrest and facilitating follicle development. In contrast, for SP group, sustained GPR3 expression maintains elevated cAMP levels, which are essential for suppressing premature meiotic resumption.

This study further shows that ovarian expression of ADCY3, ADCY5, PDE1A, and PDE3A in the striped hamster is photoperiodically regulated. Correlation analyses indicate that ADCY5, PDE1A, and PDE3A, rather than ADCY3, are the dominant contributors to photoperiod-driven modulation of intra-oocyte cAMP concentrations. Consistent with these findings, prior studies demonstrate that the LH surge triggers GPCR desensitization, resulting in adenylate cyclase (ADCY) inhibition and a consequent decrease in intra-oocyte cAMP concentrations (17). Studies show that FSH treatment *in vitro*-cultured rat granulosa cells enhances the activity of cAMP-hydrolyzing phosphodiesterases (PDEs), leading to decreased intracellular cAMP concentrations (18, 52). Genetic ablation or pharmacological inhibition of PDE1A and PDE3A elevates intra-oocyte cAMP levels (58). Moreover, *in vivo* FSH administration in rats upregulates PDE3 transcription, predominantly PDE3A, resulting in reduced intra-oocyte cAMP concentrations (59). Collectively, these findings support a coordinated regulatory model in which FSH and LH jointly modulate the opposing activities of ADCY and PDEs to fine-tune intra-oocyte cAMP homeostasis. In turn, cAMP-PKA signaling governs key processes in follicle development, including granulosa cell differentiation, estradiol biosynthesis, and oocyte meiotic competence acquisition.

In conclusion, this study identifies GPR3, ADCY5, PDE1A, and PDE3A as photoperiod-sensitive regulators of intra-oocyte cAMP homeostasis based on correlation analysis in the striped hamster. Their differentiate expression among various photoperiod suggests a tightly integrated signaling module that translates environmental light cues into precise intracellular cAMP dynamics governing follicle progression. Future functional studies, using isoform-selective inhibitors or genetic perturbation of GPR3, ADCY5, PDE1A, or PDE3A in an *in vitro* cultured follicle system, will be essential to validate causal roles and dissect hierarchical interactions within this pathway.

## 5 Conclusions

**Figure 7:**
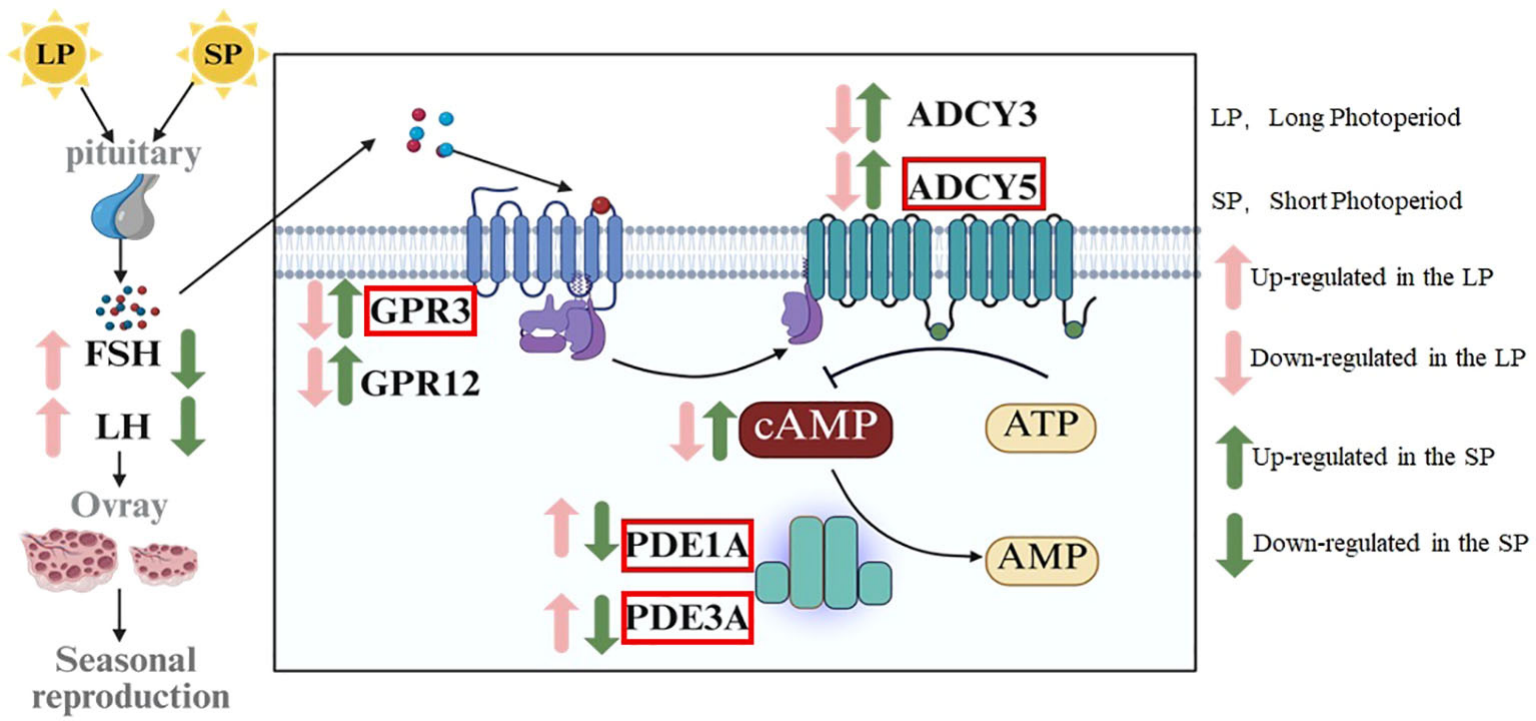
The molecular pathway underlying photoperiodic regulation of cAMP levels in the ovaries of striped hamsters. The factors marked in red boxes exert significant regulatory effects on cAMP levels in the ovaries of striped hamsters in response to photoperiod.

Photoperiod regulates the synthesis and secretion of FSH and LH via the hypothalamic-pituitary-gonadal axis (HPGA), which in turn modulate the expression of *GPR3*, *ADCY5*, *PDE1A*, and *PDE3A* in the ovaries of striped hamsters, thereby influencing intracellular cAMP levels. As a second messenger, cAMP regulates granulosa cell proliferation and apoptosis, follicle development, and ultimately reproductive activity in striped hamsters. Long photoperiod (LP) enhances the synthesis and secretion of FSH and LH. These hormonal signals subsequently downregulate *GPR3* and *ADCY5* expression, upregulate *PDE1A* and *PDE3A* expression, reduce intracellular cAMP levels, promote granulosa cell proliferation, suppress granulosa cell apoptosis, and ultimately stimulate follicle development (**Figure 7**). Short photoperiod (SP) suppresses the synthesis and secretion of FSH and LH. These hormonal signals subsequently upregulate *GPR3* and *ADCY5* expression, downregulate *PDE1A* and *PDE3A* expression, elevate intracellular cAMP levels, promote granulosa cell apoptosis, inhibit granulosa cell proliferation, and ultimately suppress follicle development (**Figure 7**). GPR3, ADCY5, PDE1A, and PDE3A may act as key regulators of follicle development in striped hamsters in response to photoperiodic cues, and these findings provide potential molecular targets for modulating reproductive activity in photoperiod-sensitive seasonal breeding species.

## Data Availability

The data presented in the study are deposited in the article/**Supplementary Material**.
