# Supplementary material for: The cAMP signaling pathway mediates photoperiod-induced follicle development in striped hamsters (Cricetulus barabensis) supported by association analyses
Source: Front Endocrinol (Lausanne). 2026 Apr 16;17:1794734. doi: 10.3389/fendo.2026.1794734 (PMC13128394; doi:10.3389/fendo.2026.1794734)
Supplement: Supplementary file 1 [file DataSheet1.docx]

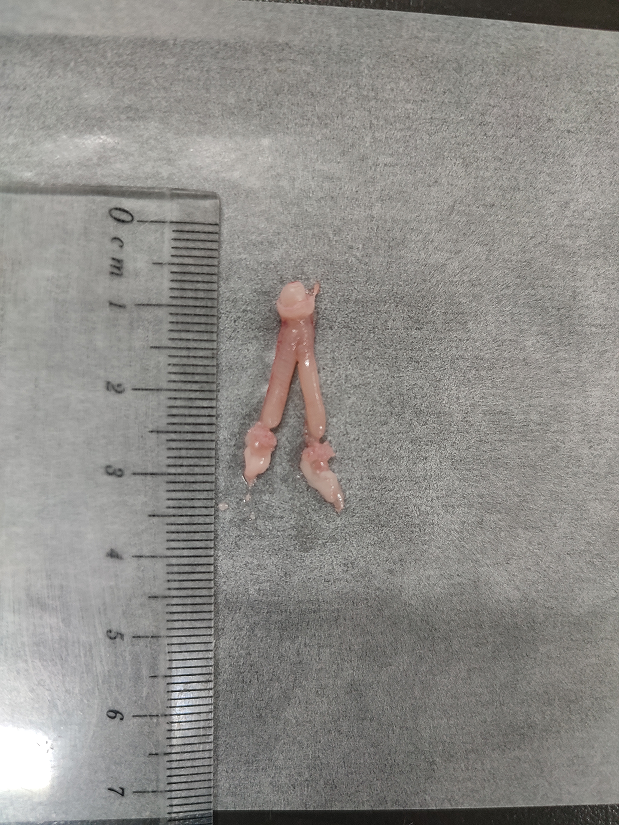

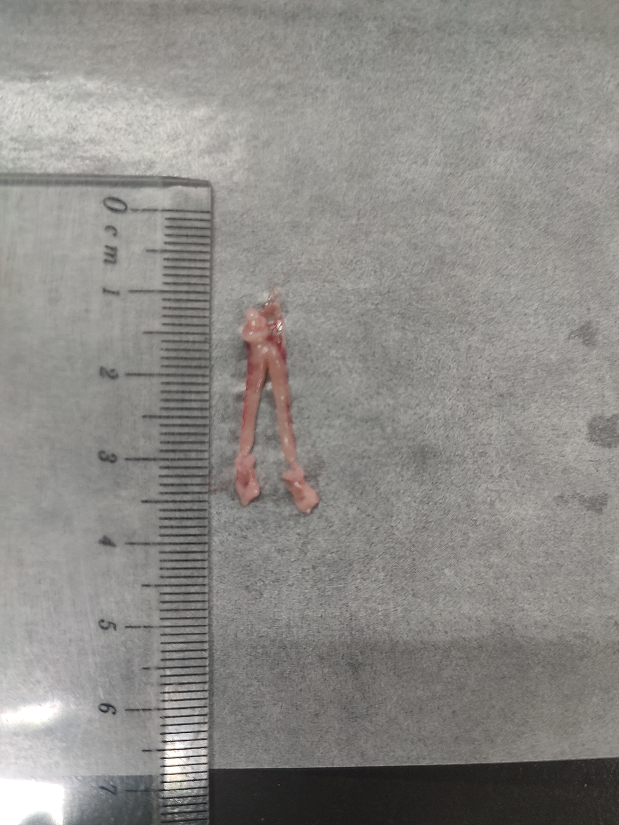

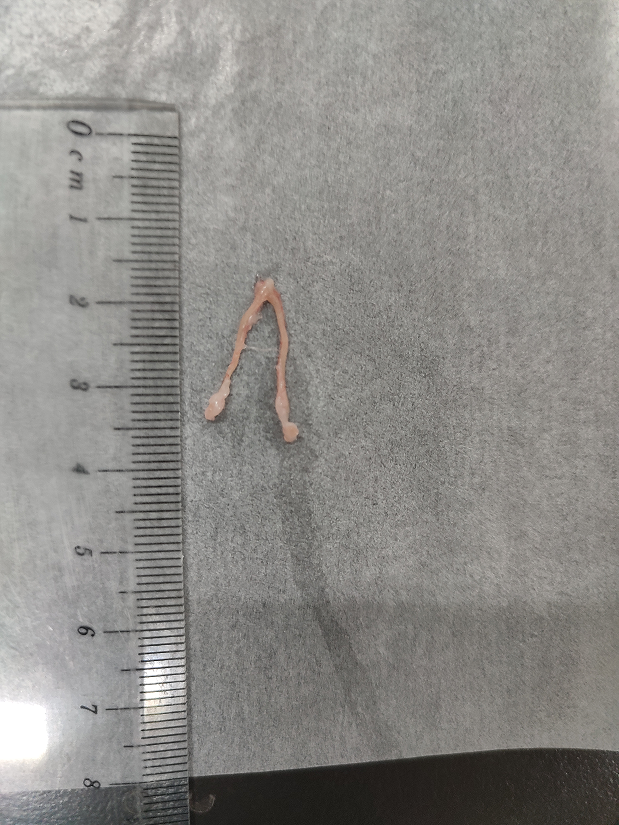


**Supplementary Figure 1 A Reproductive organs of the LP group**  **Supplementary Figure 1 B Reproductive organs of the MP group**  **Supplementary Figure 1 Reproductive organs of the SP group**


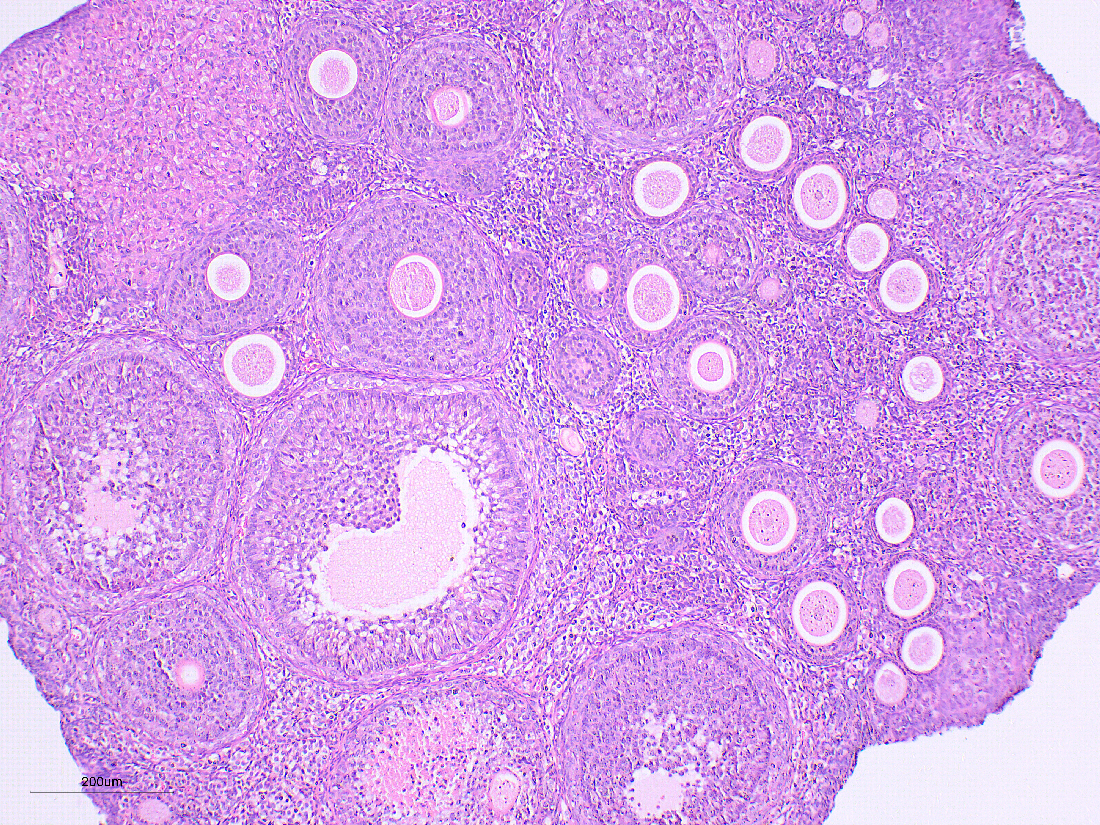

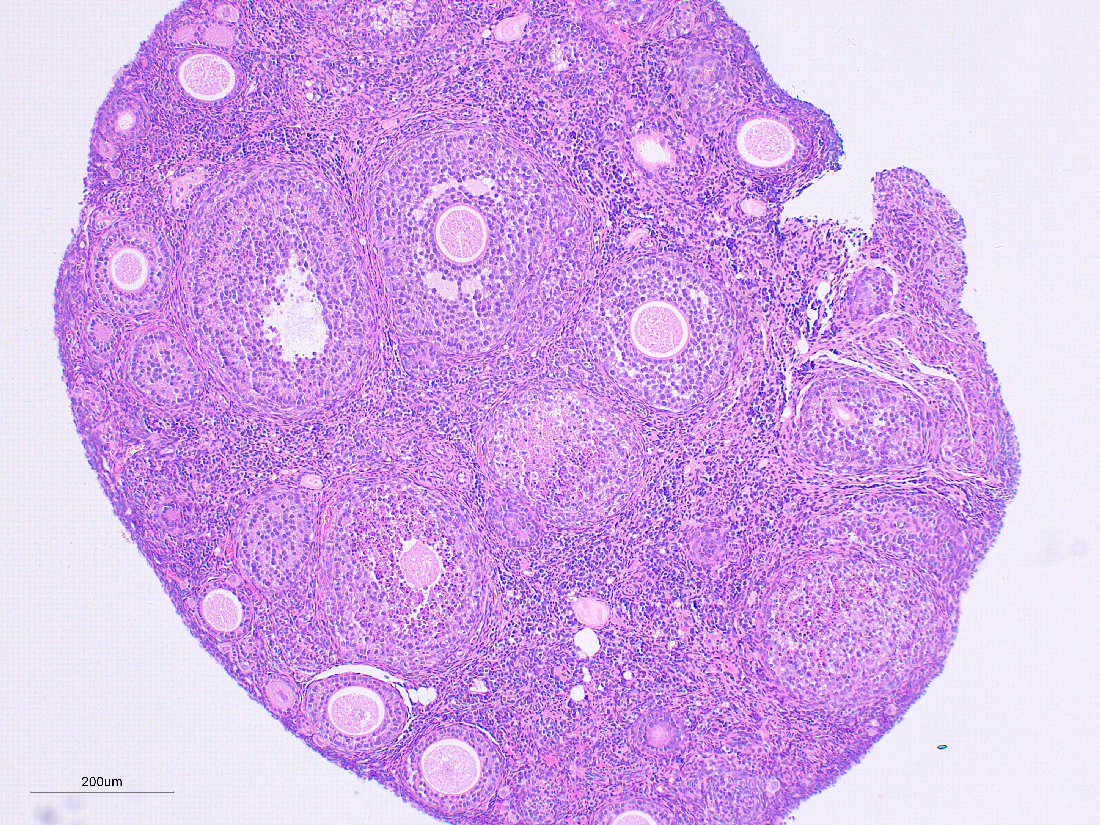


**Supplementary Figure 2 A HE staining of ovarian sections from LP groups at 10×magnification**  **Supplementary Figure 2 B HE staining of ovarian sections from MP groups at 10×magnification**


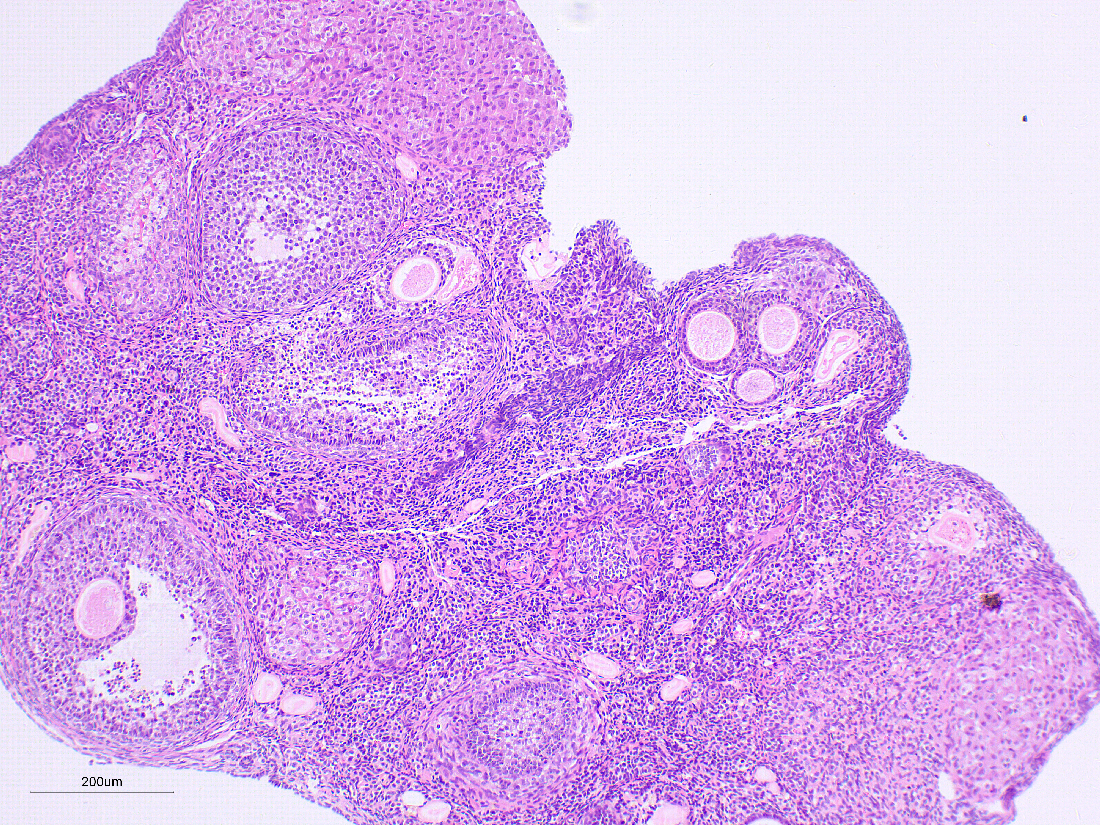


**Supplementary Figure** **2 C HE staining of ovarian sections from SP groups at 10×magnification** **Supplementary Figure 2 D HE staining of follicles at various developmental stages at 40×magnification**


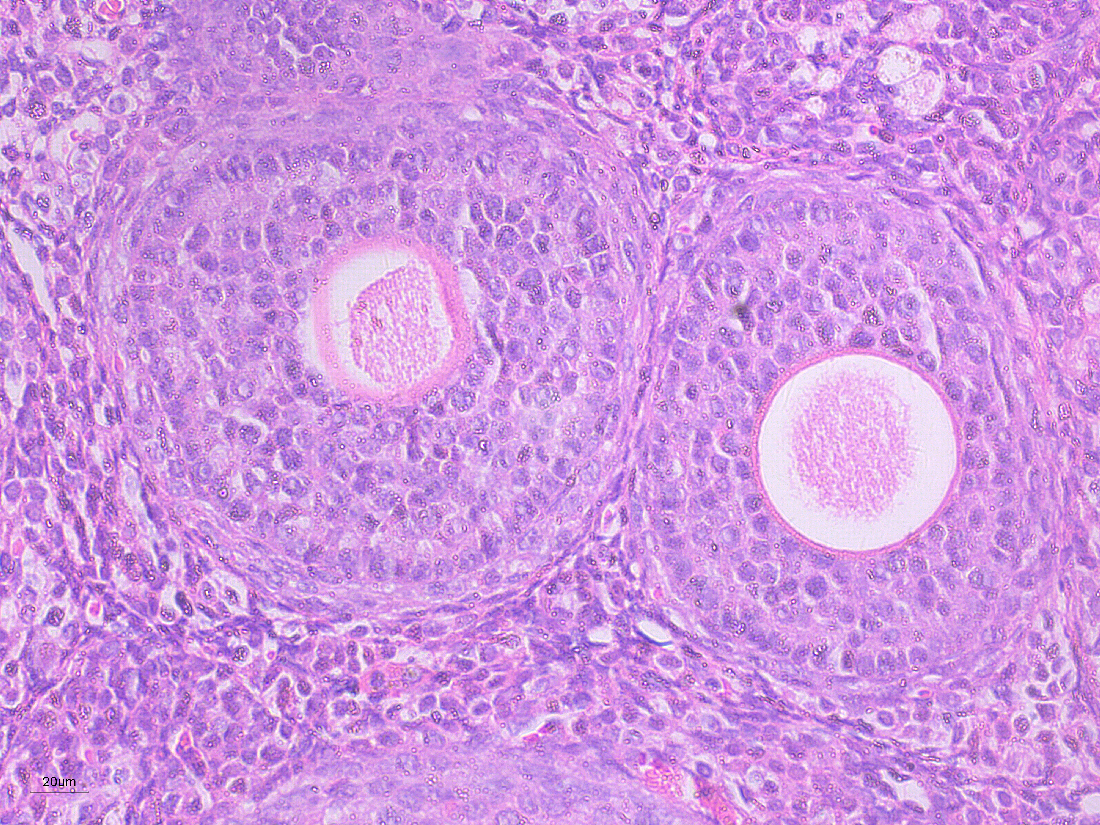


**Supplementary Figure** **2 E HE staining of follicles at various developmental stages at 40×magnification**  **Supplementary Figure 2 F HE staining of follicles at various developmental stages at 40×magnification**
